# Myeloid-Derived Suppressor Cells Induce Exhaustion-Like CD8^+^ T Cells during JEV Infection

**DOI:** 10.7150/ijbs.102372

**Published:** 2024-11-04

**Authors:** Weijia Zhang, Qing Yu, Xiaochen Gao, Haowei Chen, Jie Su, Yanru Chen, Yanling Li, Nan Zhang, Zhenfang Fu, Min Cui

**Affiliations:** 1 State Key Laboratory of Agricultural Microbiology, College of Veterinary Medicine, Huazhong Agricultural University, Wuhan, China.; 2 Key Laboratory of Preventive Veterinary Medicine in Hubei Province, The Cooperative Innovation Centre for Sustainable Pig Production, Wuhan, China.; 3 Key Laboratory of Development of Veterinary Diagnostic Products, Ministry of Agriculture of the People's Republic of China, Wuhan, China.; 4 International Research Centre for Animal Disease, Ministry of Science and Technology of the People's Republic of China, Wuhan, China.

**Keywords:** CD8^+^ T cells, Japanese encephalitis virus, MDSCs, PD-1, TIM-3

## Abstract

Japanese encephalitis (JE), caused by Japanese encephalitis virus (JEV), is a mosquito-borne zoonotic disease and a leading cause of viral encephalitis worldwide. While JEV has the ability to traverse the blood-brain barrier (BBB), the precise mechanisms by which it inhibits the immune response prior to penetrating the BBB remain unclear, presenting obstacles in the development of efficacious therapeutic interventions. This study investigated the impact of JEV on CD8^+^ T cell responses, with a particular focus on the dysfunction of CD8^+^ T cells during JEV infection. Our results demonstrated that JEV infection significantly elevated the expression of PD-1 and TIM-3 on CD8^+^ T cells, which are markers of T cell exhaustion, leading to inhibited function and impaired differentiation, resulting in a poorer prognosis in mice. Compared with nondiseased mice, symptomatic mice presented a greater proportion of exhaustion-like CD8^+^ T cells. *In vitro* experiments further demonstrated that MDSCs induced an exhaustion-like state in CD8^+^ T cells, characterized by significant upregulation of PD-1 and TIM-3 expression. Notably, blocking TIM-3 or depleting MDSCs restored CD8^+^ T cell functionality by rescuing the expression of IFN-γ and TNF-α. Furthermore, the depletion of MDSCs not only alleviated T cell exhaustion-like phenotypes but also improved survival rates in JEV-infected mice. These findings suggest that JEV promotes immune evasion through MDSC-induced CD8^+^ T cell exhaustion-like states and identify TIM-3 as a promising therapeutic target for JE treatment.

## Introduction

Japanese encephalitis (JE) is a zoonotic viral illness caused by Japanese encephalitis virus (JEV) and is transmitted via mosquito bites 1, 2. JEV is a single-stranded positive-sense RNA virus from the Flavivirus genus and the Flaviviridae family. It is closely related to dengue virus (DENV), yellow fever virus (YFV), Zika virus (ZIKV), and tick-borne encephalitis virus (TBEV) 3*.* JEV is a major cause of viral encephalitis in Asia, with approximately 68,000 cases reported each year. The increasing incidence of JEV transmission to previously untouched locations 3, 4, along with its detection in vertebrate hosts within endemic areas, underscores the growing global threat of JEV dissemination. Although most patients are clinically asymptomatic or experience mild fever, nearly 1% of those infected with JEV may develop encephalitis, a condition that can be fatal in up to 30% of cases due to the virus crossing the blood-brain barrier (BBB) 2. However, the precise mechanism responsible for the suppression of immune responses against JEV needs to be completely understood and requires a systematic explanation.

JEV has developed a variety of immune evasion mechanisms that allow it to resist clearance by the host immune system before entering the central nervous system. JEV uses a multitude of molecular pathways to decrease the interferon (IFN) response, which allows the virus to evade the host's innate immunity 5-8. Although JEV normally boosts adaptive immunity to combat infection 9, 10, current research has revealed how JEV impairs adaptive immunity. JEV inhibits the function of T follicular helper (Tfh) cells via the activation of myeloid-derived suppressor cells (MDSCs) 11, which in turn disrupts humoral immunity. Additionally, JEV suppresses the maturation of dendritic cells (DCs), leading to an increase in regulatory T cells (Tregs) and a reduction in T helper 1 (Th1) cells 12, 13. Furthermore, the antigen presentation function of DCs, which is critical for T cell responses, is impaired during JEV infection 14. Given the usual incubation period of 3-14 days observed in patients 15, it is evident that JEV has evolved multiple mechanisms to suppress adaptive immune responses, revealing the complexities of its interaction with the host immune system.

CD8^+^ T cells, which are crucial to the adaptive immune response, play an important role in managing JEV infections by directly destroying infected cells and producing antiviral substances. Adoptive transfer of virus-specific cytotoxic T lymphocytes (CTLs) provides significant protection against the fatal threats of JEV 16. In adult mice, a reduction in CD8^+^ T cells results in heightened sensitivity to JEV and simultaneous breakdown of the BBB 17, highlighting the critical function of CTLs in antiviral immunity against JEV. Individuals with subclinical JEV infection exhibit a considerably increased frequency of CD8^+^ T cells, whereas those with JEV-induced encephalitis present a deficiency in CD8^+^ T cells alongside an increase in CD4^+^ T cells. This disparity emphasizes the importance of CD8^+^ T cells in the immunological response to JEV 18.

T cell exhaustion is a dysfunctional state that occurs during viral infections and malignancies and is characterized by a progressive loss of effector activities, diminished proliferative ability, and sustained expression of inhibitory receptors such as PD-1 and TIM-3 19, 20. In chronic virus infections such as human immunodeficiency virus (HIV) and hepatitis C virus (HCV), exhausted T cells fail to control viral replication effectively, leading to persistent infection and disease progression 21, 22. Recent research has revealed that during acute viral infections, CD8^+^ T cells can also exhibit features of exhaustion, leading to reduced functional effectiveness 23-25. JEV infection has been found to increase PD-1 expression as well 11, 26. However, it is unclear whether JEV promotes immunological evasion by inducing T cell exhaustion.

This study investigated the phenotype of CD8^+^ T cells after JEV infection and revealed significant upregulation of PD-1 and TIM-3 expression on these cells. Based on the expression of these molecules, CD8^+^ T cells were divided into three subpopulations: PD-1^-^TIM-3^-^, PD-1^+^TIM-3^-^, and PD-1^+^TIM-3^+^ cells. Notably, the PD-1^+^TIM-3^+^ cells were defined as exhaustion-like CD8^+^ T cells due to their elevated inhibitory receptor expression and diminished cytokine production. Furthermore, a link was discovered between the proportion and function of PD-1^+^TIM-3^+^CD8^+^ T cells and illness progression in mice. Additionally, JEV-induced MDSCs can inhibit CD8^+^ T cell proliferation and contribute to the development of an exhaustion-like state in CD8^+^ T cells both *in vivo* and *in vitro*. Crucially, inhibiting TIM-3 or decreasing the number of MDSCs *in vivo* restored CD8^+^ T cell function and improved animal survival. These findings highlight the involvement of MDSC-induced exhaustion-like CD8^+^ T cells in JEV immunological evasion and suggest that TIM-3 may be a promising therapeutic target for JEV treatment.

## Methods and materials

### Mice and viruses

Adult female C57BL/6 mice aged 6-8 weeks were purchased from the Laboratory Animal Services Centre (Huazhong Agricultural University) and were cared for in accordance with the Committee for Protection, Supervision, and Control of Experiments on Animals at Huazhong Agricultural University. The pathogenic strain JEV-P3 and the vaccine strain JEV-SA14-14-2 were stored in our laboratory. To amplify the virus, suckling mice were injected with 1×10^3^ plaque-forming units (PFUs) of JEV in 10 µl of Dulbecco's modified Eagle's medium (DMEM). The infected mice were subsequently euthanized, and the brains were extracted from the deceased. The whole brain tissue of the suckling mice was homogenized in a volume of DMEM equal to ten times the weight of the brain tissue, with homogenization conducted on ice. The homogenate was subjected to centrifugation at 7,000 × g for 45 min at 4°C. After centrifugation, the supernatants were carefully separated and stored at -80°C. The viral titer of JEV was evaluated via plaque assays performed with the baby hamster kidney fibroblast line BHK-21 in accordance with previously established procedures 27.

### Cell lines

BHK-21 and EL-4 thymoma cells were previously maintained in our laboratory. The cell lines were grown in DMEM supplemented with 10% FBS, 100 U/ml penicillin, and 100 μg/ml streptomycin at 37°C in a 5% CO_2_ environment.

### Mouse model of JEV infection

On day 0, the specified PFUs of JEV (strain P3 or SA14-14-2) were administered intravenously (i.v.) into mice in 100 μl of DMEM. The control animals received the same amount of DMEM in the same way. In the 21-day monitoring experiment, infection with strain P3 was carried out using 5×10^4^ PFUs, and every 7 days, three or more JEV-infected mice were sacrificed for flow cytometry analysis, continuing until 21 days post infection (dpi). In all other experiments, 1×10^5^ PFUs were used. At designated time points, samples were collected for flow cytometry analysis or RNA extraction.

### Flow cytometry

Single-cell suspensions from the mouse spleen, lymph node, blood, and cultured cells were preincubated for 10 min at room temperature with 0.3 μg of anti-CD16/CD32 antibody (BioXcell).

To stain for cell surface markers, the samples were treated with mAbs conjugated with fluorochromes in PBS containing 1% BSA at 4°C for 30 min. The cells were washed twice with 0.2% BSA in PBS before being examined via Cytoflex (Beckman Coulter) flow cytometers and sorted via MoFlo XDP. The following monoclonal antibodies conjugated with fluorophores were used:

APC-conjugated anti-Gr-1 (RB6-8C5; BioLegend), PerCP-Cy5.5-conjugated anti-CD11b (M1/70; BioLegend), PerCP-Cy5.5-conjugated anti-CD3ε (145-2C11; BioLegend), Pacific Blue-conjugated anti-CD8a (53-6.7; BioLegend), PE-conjugated anti-CD366 (RMT3-23; BioLegend), APC-conjugated anti-CD366 (RMT3-23; BioLegend), FITC-conjugated anti-CD279 (29F.1A12; BioLegend), and PE-conjugated anti-IL-21r (4A9; BioLegend) antibodies were used.

To analyze cytokine production, the cells were restimulated *in vitro* for 5 h using a Cell Activation Cocktail with Brefeldin A (BioLegend) at 37°C in a 5% CO_2_ environment. After being stained with surface markers, the cells were fixed and permeabilized with a fixation/permeabilization kit (BD Biosciences) for 20 min at 25°C. They were then stained with PE-conjugated anti-IL-2 (JES6-5H4; BioLegend), PE-conjugated anti-IFN-γ (XMG1.2; BioLegend), PE-conjugated anti-TNF-α (MP6-XT22; BioLegend), and PE-conjugated anti-granzyme B (GB11; BioLegend), PE-conjugated anti-perforin (S16009A; BioLegend), and PE-conjugated anti-CD107a (1D4B; BioLegend) for 40-60 min at 4°C. Finally, the number of cells was determined via flow cytometry. The proliferation of CD8^+^ T cells was measured via intracellular labeling with FITC-Ki-67 (7B11; Invitrogen).

### Quantitative real-time PCR and gene expression profiling

Following the manufacturer's protocol, total RNA was extracted from the tissue via TRIpure reagent (Adilab). cDNA templates were synthesized with ABScript Neo RT Master Mix for qPCR with gDNA Remover (ABclonal). Real-time PCR was performed via a QuantStudio 5 real-time PCR instrument (Applied Biosystems) with the Genious SYBR Green Fast qPCR Mix (ABclonal). The mRNA levels of the target genes were normalized to β-actin expression.

### *In vitro* coculture assays

CD8^+^ T cells were purified from the spleens of C57BL/6 mice via the MojoSort™ Mouse CD8^+^ T cell Isolation Kit (BioLegend) and labeled with 2.5 μM CFSE for 10 min at 25°C. The cells were seeded in 96-well plates (1×10^5^ cells per well) in complete RPMI 1640 media and stimulated with plate-coated anti-CD3 (5 μg/ml) and anti-CD28 Abs (2 μg/ml). MDSCs (CD11b^+^Gr-1^+^) were sorted from P3-infected mice and cultivated with various ratios of CD8^+^ T cells in the presence of TIM-3 mAb (RMT3-23; BioXcell) or anti-IgG2a Ab (BioXcell) for 4 days at 37°C in a 5% CO_2_ environment.

### *In vitro* CTL assays

C57BL/6 mice infected with JEV were immunized with OVA at 1 dpi, and *in vitro* CTL killing activities were measured on day 7. To conduct an *in vitro* cytotoxicity test, EL-4 lymphoma cells (1×10^4^) were pulsed with 2 μM OVA peptide (257-264) for 1 h and cocultured with CD8^+^ T cells obtained from OVA-immunized mice at various ratios for 12 h. After incubation, the cells were collected, labeled with 0.1 μg of DAPI (Sigma‒Aldrich), and analyzed by flow cytometry.

### Blockade of TIM-3 *in vivo*

The mice were intraperitoneally (i.p.) injected with 100 μg of mAb specific for TIM-3 (BioXcell) or a rat IgG2a isotype control Ab (BioXcell) per mouse 1 day prior to P3 infection followed by injections every 2 days until 5 dpi.

### Elimination of MDSCs *in vivo*

The use of all-trans retinoic acid (ATRA) to eradicate MDSCs *in vivo* was consistent with earlier studies 28. ATRA (Sigma‒Aldrich) was given i.p. (20 mg/kg/d) starting 3 days before P3 infection and every 12 h until day 5. ATRA was dissolved at 40 mg/ml in dimethyl sulfoxide (DMSO) and diluted with solvent.

### ELISPOT of IFN-γ

ELISPOT experiments were performed by adding the JEV peptide to the spleen cells of IgG2a-treated P3-infected mice and α-TIM-3-treated P3-infected mice on IFN-γ antibody-precoated plates (Dakewe, China). Splenocytes treated with medium alone were used as a negative control, while those stimulated with PMA and ionomycin were used as positive controls. The plates were incubated at 37°C in a 5% CO_2_ atmosphere for 24 h. The cells were lysed according to the manufacturer's specifications, and the plates were washed six times with washing buffer. Biotinylated IFN-γ antibodies were added, and the samples were incubated for 1 h at 37°C. After the plates were washed six times to eliminate any unbound antibodies, streptavidin-HRP was added, and the mixture was incubated for an additional hour at the same temperature. Afterward, the plates were treated with AEC buffer and incubated in the dark at room temperature for 15 min. Finally, the reaction was stopped, and the plates were air-dried. Positive spots were quantified via a Mabtech IRIS^TM^ automatic plate reader (Mabtech).

### Ethics statement

All animal experiments were approved by the Huazhong Agricultural University Research Ethics Committee (HZAUMO-2019-060) and carried out in accordance with the guidelines established by the Committee for Protection, Supervision, and Control of Animal Experiments at Huazhong Agricultural University, Hubei, China.

### Statistical analysis

All the experiments were performed in triplicate. The data were analyzed via GraphPad Prism 7.0 (GraphPad, La Jolla, CA, USA), and* p*-values were calculated via a two-tailed, unpaired* t*-test for means ± SEMs. One-way ANOVA was used to assess data from multiple groups.

## Results

### Increased expression of TIM-3 and PD-1 on CD8^+^ T cells after JEV infection

**Figure 1:**
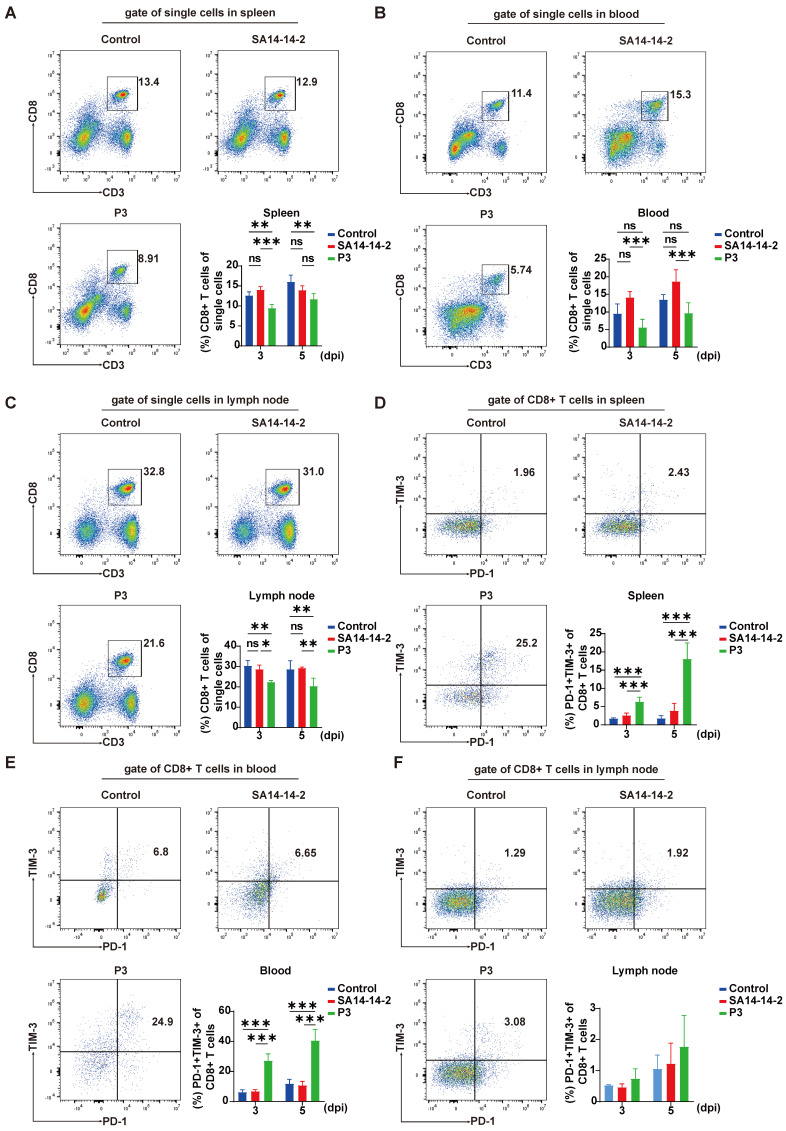
** P3 alters the proportions and coinhibitory molecule expression of CD8^+^ T cells in the spleen, peripheral blood, and lymph nodes.** C57BL/6 mice (female, 6-8 weeks old) were i.v. injected with 10^5^ PFUs of JEV P3 or SA14-14-2, and cells isolated from the spleen, peripheral blood and lymph nodes were analyzed at 3 dpi and 5 dpi via flow cytometry. **(A, B, C)** Flow cytometry plots and frequencies of CD8^+^ T cells in the spleen, peripheral blood and lymph nodes of control, SA14-14-2, and P3-infected mice. **(D, E, F)** Flow cytometry plots and frequencies of PD-1^+^TIM-3^+^ cells within CD8^+^ T cells in the spleen, peripheral blood and lymph nodes of control, SA14-14-2, and P3-infected mice. The data are presented as arithmetic means ± SEMs of three experiments from one participant at each time point (n = 4 for the control group and n = 4 for the infection group). ns, not significant; *, *P* < 0.05; **, *P* < 0.01; ***, *P* < 0.001.

A previous study revealed a link between JEV infection and a decrease in the percentage of CD4^+^ T cells, as well as decreased activation of CD4^+^ T cells in the early stages of JEV infection in mice. To investigate the impact of JEV infection on CD8^+^ T cells, mice were i.v. injected with the JEV P3 strain (1×10^5^ PFUs) or the vaccine strain SA14-14-2 (1×10^5^ PFUs). The proportions of CD8^+^ T cells in different immune organs of JEV-infected mice were analyzed via flow cytometry. The number of splenic CD8^+^ T cells was significantly reduced at 3 and 5 dpi in JEV P3 but not in SA14-14-2 infected mice (Figure 1A). Since JEV primarily replicates in the lymph nodes, leading to enlarged lymph nodes in patients 29, we aimed to evaluate whether the loss of splenic CD8^+^ T cells was due to lymphocyte recirculation to the lymph nodes. Flow cytometry analysis was performed, and the results revealed that the CD8^+^ T cell proportions decreased in both the peripheral blood and the lymph nodes at 3 and 5 days after JEV P3 infection (Figure 1B & 1C).

After acute infection, PD-1 expression is detected in antigen-specific T cells 30. Similarly, during dengue virus infection, activated antigen-specific T cells exhibit increased PD-1 expression 31. However, in the context of chronic antigen stimulation, TIM-3 is commonly coexpressed with PD-1, indicating a state of CD8^+^ T cell exhaustion 32, 33. Considering that PD-1 expression is associated with acute antigen-specific T cell activation, while the coexpression of PD-1 and TIM-3 signifies CD8^+^ T cell exhaustion during chronic antigen stimulation, we assessed the expression of PD-1 and TIM-3 on CD8^+^ T cells at 3 and 5 dpi to investigate the potential occurrence of T cell exhaustion during JEV infection. We observed a significantly greater frequency of PD-1^+^TIM-3^+^CD8^+^ T cells in both the spleen and peripheral blood of JEV P3-infected mice compared to the SA14-14-2 group (Figure 1D & 1E). In contrast, there was no substantial upregulation of these dual-positive cells in the lymph nodes (Figure 1F). Additionally, PD-1^+^TIM-3^-^CD8^+^ T cells were upregulated in all tissues examined (Supplementary Figure 1). Although TIM-3^+^PD-1^-^CD8^+^ T cells were modestly increased in the spleen, their proportions were significantly lower than those of the PD-1^+^TIM-3^+^ cells. These findings indicate that JEV infection not only reduces the number of CD8^+^ T cells but also results in the production of PD-1^+^TIM-3^+^CD8^+^ T cells, which may be exhaustion-prone CD8^+^ T cells.

### Coexpression of PD-1 and TIM-3 is associated with dysfunction of CD8^+^ T cells

**Figure 2:**
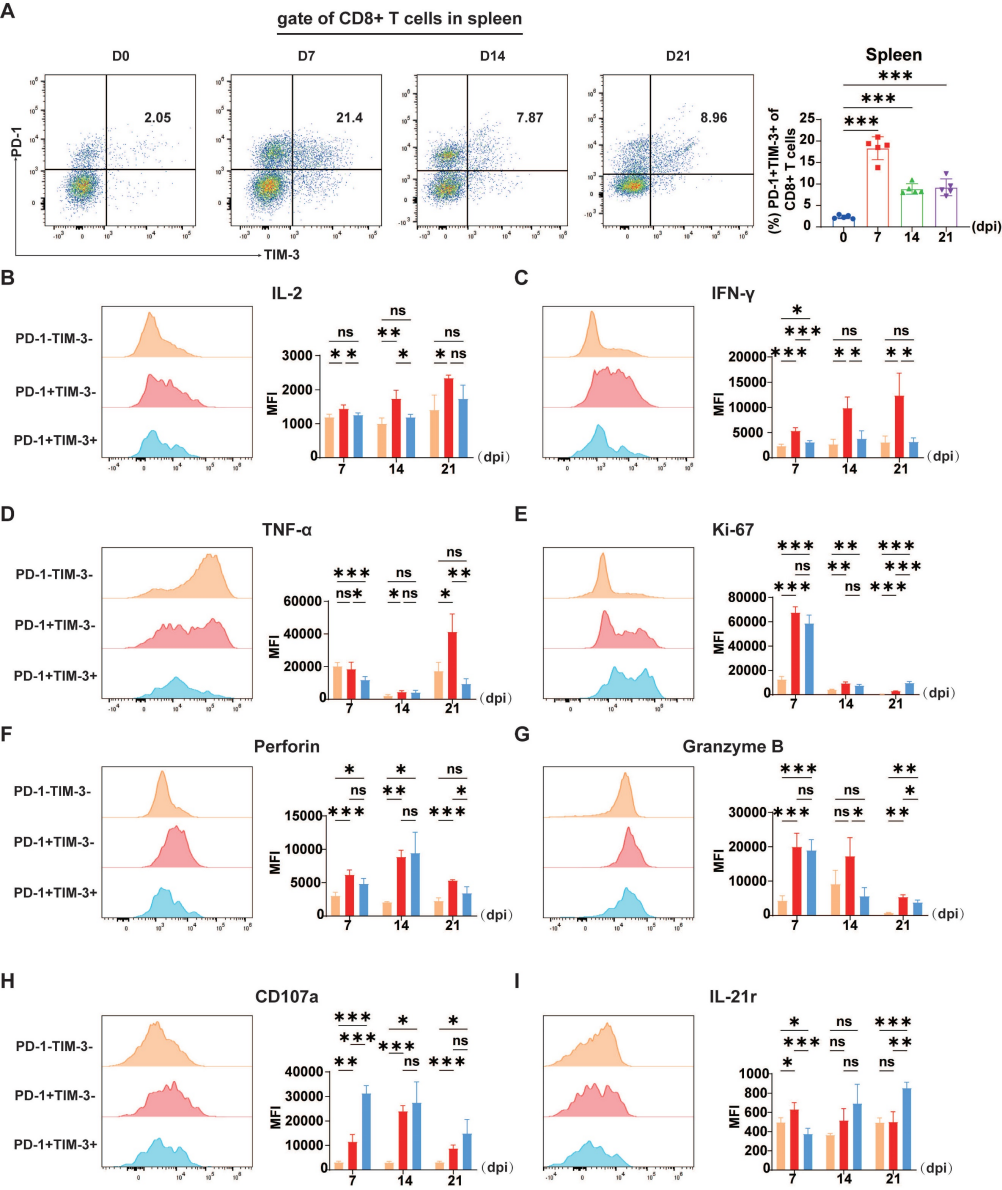
** Proliferation and cytokine production in CD8^+^ T cells during P3 infection.** C57BL/6 mice (female, 6-8 weeks old) were i.v. injected with 5×10^4^ PFUs of P3, and splenocytes were analyzed every 7 days from 0-21 dpi via flow cytometry. **(A)** Flow cytometry plots and frequencies of PD-1^+^TIM-3^+^ cells within CD8^+^ T cells in the spleen during P3 infection. **(B, C, D, E, F, G, H)** Representative flow cytometry histograms (left) and MFI (right) of IL-2, IFN-γ, TNF-α, Ki-67, perforin, granzyme B, and CD107a staining in different CD8^+^ T cell subpopulations are shown. **(I)** A representative flow cytometry histogram (left) and MFI (right) of IL-21r staining of different CD8^+^ T cells subpopulations are shown. Representative results from repeated experiments are shown. The data are presented as arithmetic means ± SEMs. ns, not significant; *, *P* < 0.05; **, *P* < 0.01; ***, *P* < 0.001.

Generally, T cell exhaustion is a result of prolonged exposure to stimuli. Thus, we conducted sustained observation of exhaustion-like CD8^+^ T cells throughout a 21-day period following JEV infection. A persistent enrichment of these exhaustion-like CD8^+^ T cells was observed throughout the 21-day post-infection period, with a peak at 7 dpi (Figure 2A). Nonetheless, T cell exhaustion is defined by the loss of T cell effector capabilities, such as cytokine production, diminished proliferative capability and cell death *in vitro* 34.

To verify whether TIM-3^+^ cells exhibit more serious dysfunction, we categorized CD8^+^ T cells into three subsets according to PD-1 and TIM-3 expression: PD-1^-^TIM-3^-^, PD-1^+^TIM-3^-^, and PD-1^+^TIM-3^+^. Subsequently, we compared the intracellular expression of proinflammatory cytokines IFN-γ, TNF-α, and IL-2, as well as effector molecules such as granzyme B, perforin, and CD107a, which are crucial for the cytotoxic activity of CD8^+^ T cells, across these three groups to determine whether PD-1^+^TIM-3^+^ cells exhibit exhaustion-like characteristics.

JEV P3 infection significantly altered the cytokine expression patterns of the three CD8^+^ T cell subsets. Compared with PD-1^+^TIM-3^-^CD8^+^ T cells, PD-1^+^TIM-3^+^CD8^+^ T cells presented a notable decrease in IL-2 expression (Figure 2B). Furthermore, they produced markedly lower levels of IFN-γ and TNF-α compared to PD-1^+^TIM-3^-^ cells (Figure 2C & 2D). Additionally, Ki-67 is commonly used as a marker for actively proliferating cells, while T cell exhaustion is typically associated with a reduction in proliferative potential, our findings suggest a more complex dynamic. Although the MFI of Ki-67 was similar between PD-1^+^TIM-3^-^ and PD-1^+^TIM-3^+^ CD8^+^ T cells (Figure 2E), the percentage of Ki-67-positive cells was significantly lower in the PD-1^+^TIM-3^+^ subset compared to PD-1^+^TIM-3^-^ cells (Supplementary Figure 2A). And most proliferating cells were PD-1^+^TIM-3^-^CD8^+^ T cells (Supplementary Figure 2B), suggesting that fewer PD-1^+^TIM-3^+^ cells were actively proliferating. Since the virus-induced cytotoxicity of CD8^+^ T cells is mediated primarily by perforin and granzymes, we next evaluated whether TIM-3 expression affected their cytolytic capacity. During the initial phase of JEV infection at 7 dpi, the expression of TIM-3 did not significantly affect the levels of perforin or granzyme B (Figure 2F & 2G). However, at 14 and 21 dpi, granzyme B expression levels were significantly lower in PD-1^+^TIM-3^+^CD8^+^ T cells than in PD-1^+^TIM-3^-^CD8^+^ T cells (Figure 2G). At 21 dpi, perforin expression followed a similar trend (Figure 2F). CD107a expression is used as a marker of degranulation in CD8^+^ T cells after activation. In contrast to the findings for granzyme B and perforin, PD-1^+^TIM-3^+^CD8^+^ T cells had an improved potential for degranulation at 7 dpi (Figure 2H). Previous studies have indicated that the absence of IL-21 leads to compromised functionality of T cells in tumors and chronic viral infection 35, 36. Notably, IL-21 expression is markedly decreased in the initial phases of JEV infection 11, which could negatively affect the proper functioning of T cells. In line with this, the comparison of IL-21 receptor (IL-21r) expression across different CD8^+^ T cell subpopulations revealed that at the early time point (7 dpi), PD-1^+^TIM-3^+^CD8^+^ T cells presented significantly lower IL-21r expression than their PD-1^+^TIM-3^-^ counterparts did, suggesting a diminished capacity for IL-21-mediated signaling (Figure 2I). In conclusion, these findings show that the presence of exhaustion-like CD8^+^ T cells after JEV infection is associated with significant functional impairments, such as reduced cytokine production, while their degranulation functions remain unaffected during the early stages of infection.

### Suppressed early activation of PD-1^+^TIM-3^+^CD8^+^ T cells following JEV infection

**Figure 3:**
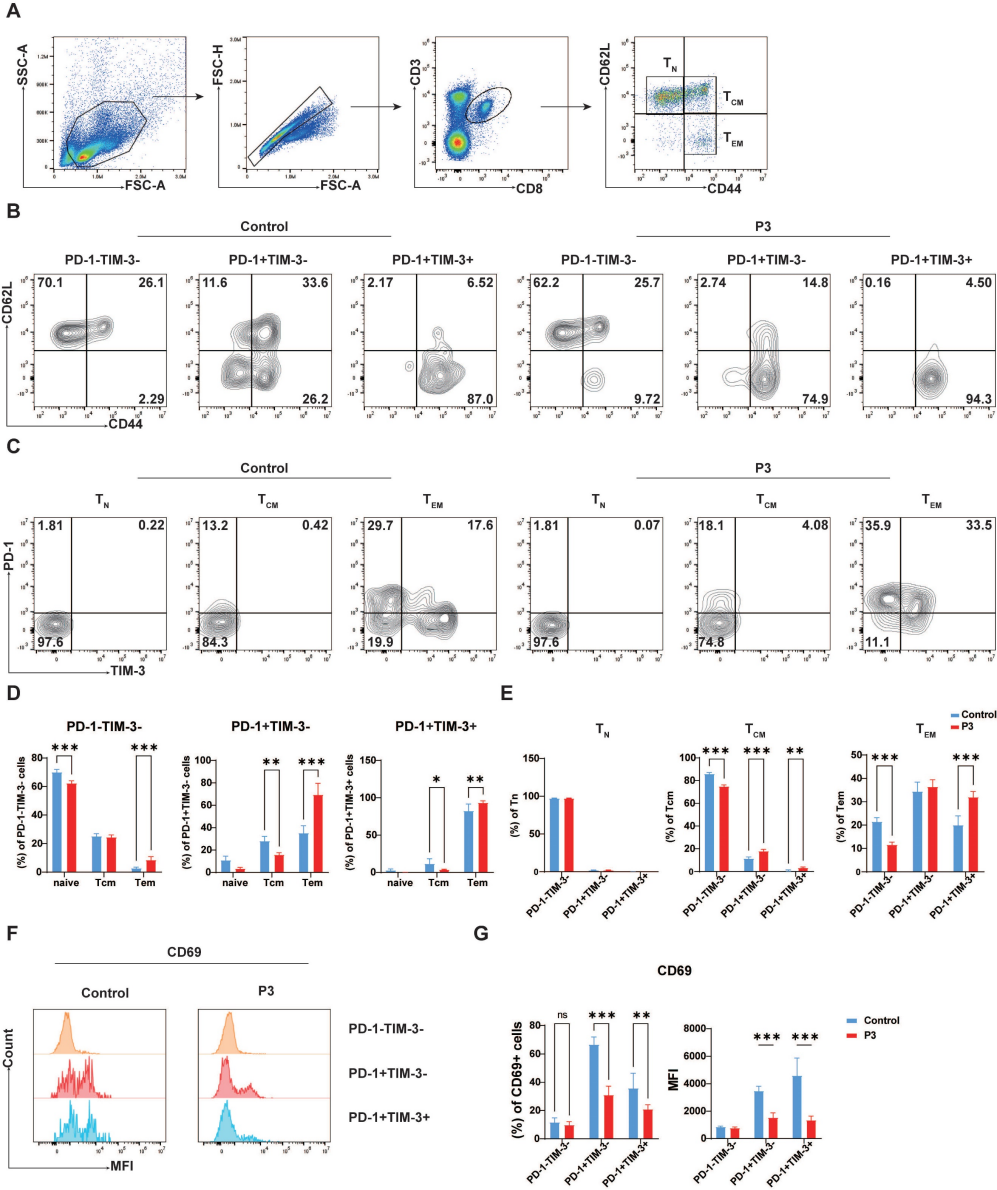
** CD8^+^ T cells coexpressing TIM-3 and PD-1 display a T cell exhaustion-like phenotype.** C57BL/6 mice (female, 6-8 weeks old) were i.v. injected with 10^5^ PFUs of P3, and splenocytes were analyzed at 7 dpi. **(A)** Flow cytometry gating strategy for determining the Tn, Tcm and Tem of CD8^+^ T cells in the spleen. **(B)** CD8^+^ T cells from control and P3-infected mice were stained with antibodies against CD44 and CD62L to determine their differentiation phenotype. Representative flow cytometry plots show the proportions of Tn, Tcm, and Tem within the PD-1^-^TIM-3^-^, PD-1^+^TIM-3^-^, and PD-1^+^TIM-3^+^ cell populations. **(C)** CD8^+^ T cells from control and P3-infected mice were stained with antibodies against CD44 and CD62L to determine their differentiation phenotype. Representative flow cytometry plots show the proportions of PD-1^-^TIM-3^-^, PD-1^+^TIM-3^-^, and PD-1^+^TIM-3^+^ cells within Tn, Tcm, and Tem populations. **(D)** Frequencies of PD-1^-^TIM-3^-^, PD-1^+^TIM-3^-^, and PD-1^+^TIM-3^+^ cells within each differentiation population. **(E)** Frequencies of Tn, Tcm, and Tem within the PD-1^-^TIM-3^-^, PD-1^+^TIM-3^-^, and PD-1^+^TIM-3^+^ cell populations. **(F)** CD8^+^ T cells from control and P3-infected mice were stained with antibodies against PD-1, TIM-3, and CD69. Representative flow cytometry histograms of CD69 expression in different subpopulations. **(G)** The frequencies and MFI of CD69^+^ cells within PD-1^-^TIM-3^-^, PD-1^+^TIM-3^-^, and PD-1^+^TIM-3^+^ cell populations were compared. Representative results from repeated experiments are shown. The data are presented as arithmetic means ± SEMs. ns, not significant; *, *P* < 0.05; **, *P* < 0.01; ***, *P* < 0.001.

In light of the observed inhibitory effect of JEV infection on T cell memory function (unpublished data), our study sought to investigate the impact of PD-1 and TIM-3 expression on the differentiation of T cell effector and memory functions. CD8^+^ T cells were classified into naive T cells (Tn, CD44^-^CD62L^+^), central memory T cells (Tcm, CD44^+^CD62L^+^), and effector memory T cells (Tem, CD44^+^CD62L^-^) on the basis of CD44 and CD62L expression (Figure 3A). Compared with those in the control group, PD-1^+^TIM-3^-^CD8^+^ T cells largely appeared as Tem (74.9%) upon JEV infection but also retained the ability to develop into Tcm (14.8%). Conversely, PD-1^+^TIM-3^+^CD8^+^ T cells were recognized almost exclusively as Tem (94.3%), with a lower frequency of Tcm (4.5%), indicating a major role in effector functions without strong memory potential (Figure 3B & 3D). However, the expression patterns of PD-1 and TIM-3 vary across different differentiation states of CD8^+^ T cells. Naive T cells did not express PD-1 or TIM-3 (Figure 3C & 3E left), which is consistent with previous research suggesting that coinhibitory molecules are often present in activated T cells. After infection with JEV, PD-1 expression was upregulated in Tcm, whereas TIM-3 expression remained low (Figure 3C & 3E middle). The distributions of PD-1^+^TIM-3^-^ and PD-1^+^TIM-3^+^ subsets among Tem were nearly equal (Figure 3C & 3E right). Additionally, CD69, a marker of early CD8^+^ T cell activation, is known to be expressed at increased levels following JEV infection 9. However, in the specific subpopulations of PD-1^+^TIM-3^-^ and PD-1^+^TIM-3^+^ CD8^+^ T cells, CD69 expression was found to be decreased (Figure 3F & 3G). Furthermore, CD69 expression in PD-1^+^TIM-3^+^ cells was lower than that in PD-1^+^TIM-3^-^ cells (Figure 3F & 3G). These findings imply that both PD-1^+^TIM-3^-^ and PD-1^+^TIM-3^+^ CD8^+^ T cells play important roles in effector responses following JEV infection, with PD-1^+^TIM-3^+^ cells exhibiting lower effector potential than PD-1^+^TIM-3^-^ cells.

### Increased exhaustion-like T Cell phenotypes in symptomatic mice

**Figure 4:**
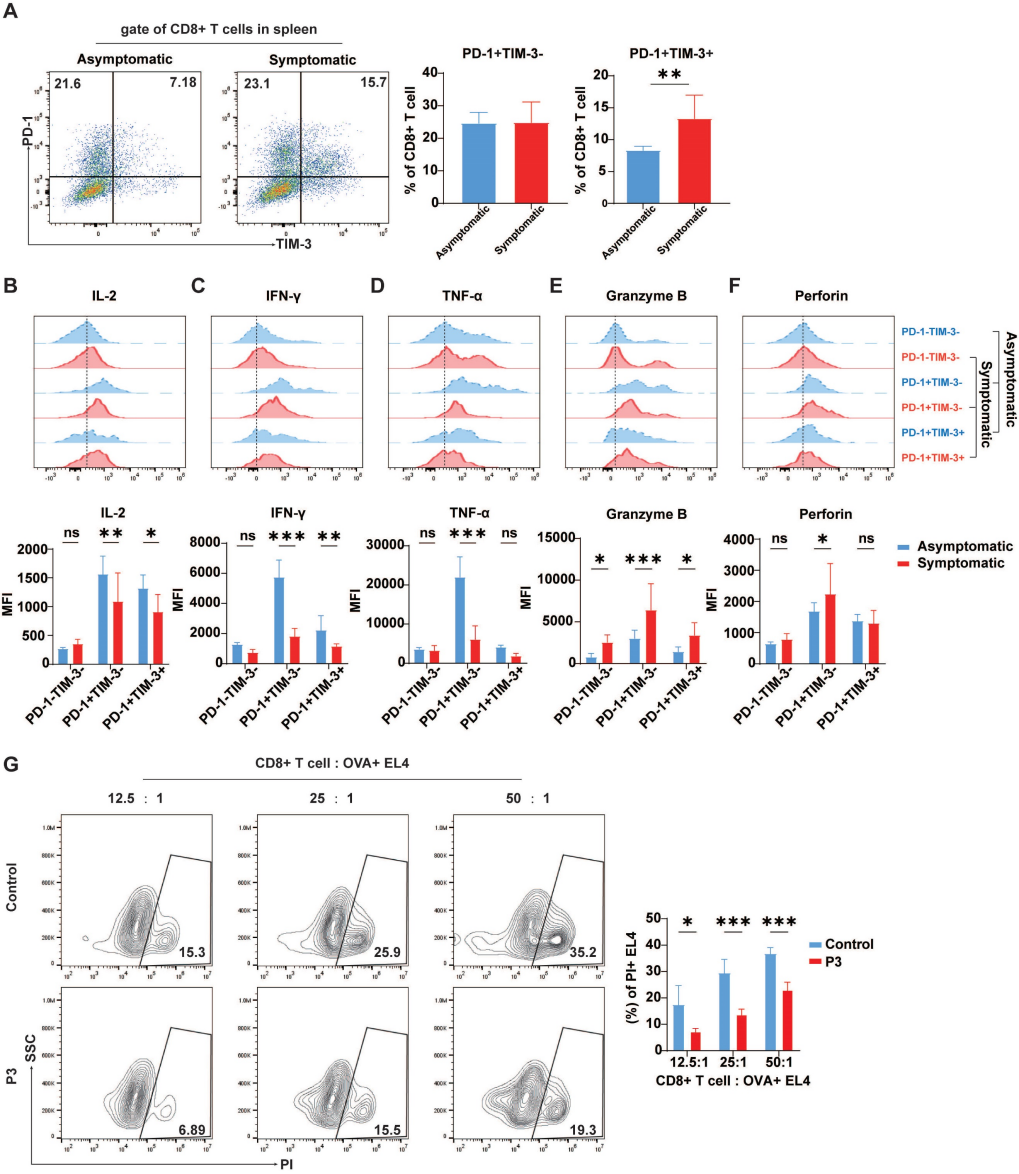
** Cytokine production in CD8^+^ T cells from asymptomatic and symptomatic mice infected with P3.** C57BL/6 mice (female, 6-8 weeks old) were i.v. injected with 10^5^ PFUs of P3, and splenocytes were analyzed at 7 dpi. **(A)** Flow cytometry plots and frequencies of PD-1^+^TIM-3^-^ and PD-1^+^TIM-3^+^ cells within CD8^+^ T cells in the spleens of asymptomatic and symptomatic mice infected with P3. **(B, C, D, E, F)** Representative flow cytometry histograms (up) and MFI (down) of IL-2, IFN-γ, TNF-α, granzyme B, and perforin staining in different CD8^+^ T cell subpopulations are shown. **(G)** C57BL/6 female mice (6-8 weeks old) infected with JEV were immunized with OVA at 1 dpi. The CD8^+^ cytotoxic activity of the mice was analyzed at 7 dpi by measuring the specific killing of OVA peptide-loaded EL-4 cells at effector-to-target ratios of 12.5:1, 25:1, and 50:1. Representative results from repeated experiments are shown. The data are presented as arithmetic means ± SEMs. ns, not significant; *, *P* < 0.05; **, *P* < 0.01; ***, *P* < 0.001.

The relationship between high TIM-3 expression and prognosis in many forms of cancer remains debatable. While in some solid tumor models, increased TIM-3 expression is linked with poor prognosis, in other malignancies, TIM-3 expression on tumor-infiltrating lymphocytes is correlated with a positive prognosis 37. Therefore, the correlation between exhaustion-like T cells and the onset of disease in JEV-infected mice was assessed by comparing the expression of TIM-3 and PD-1 on CD8^+^ T cells, along with the expression of effector molecules in the spleens of symptomatic and asymptomatic mice. Following JEV infection, the proportion of PD-1^+^TIM-3^+^CD8^+^ T cells in symptomatic mice was significantly greater than that in asymptomatic mice; while the proportion of PD-1^+^TIM-3^-^ cells remained unaffected (Figure 4A). In symptomatic mice, IL-2 expression in PD-1^+^TIM-3^-^CD8^+^ T cells was significantly lower than that in asymptomatic mice, and IL-2 expression in PD-1^+^TIM-3^+^CD8^+^ T cells was also reduced compared to asymptomatic mice (Figure 4B). IFN-γ expression was identical to that of IL-2. In symptomatic mice, IFN-γ expression levels were significantly lower in both PD-1^+^TIM-3^-^ and PD-1^+^TIM-3^+^ CD8^+^ T cells than in asymptomatic mice. There was no significant difference in IFN-γ production in PD-1^-^TIM-3^-^CD8^+^ T cells across the two groups (Figure 4C). TNF-α expression differed significantly between symptomatic and asymptomatic mice in PD-1^+^TIM-3^-^CD8^+^ T cells but not in PD-1^+^TIM-3^+^ cells (Figure 4D). However, unlike the expression of inflammatory cytokines, the expression of granzyme B in all three subsets of CD8^+^ T cells was significantly greater in symptomatic mice than in asymptomatic mice (Figure 4E), possibly due to more severe viremia in mice with symptoms and related immunopathological damage. The expression of perforin, similar to that of granzyme B, was paradoxically greater in PD-1^+^TIM-3^-^CD8^+^ T cells from symptomatic mice than in those from asymptomatic mice (Figure 4F). This imbalance between inflammatory and cytotoxic responses suggests that the heightened cytotoxic activity might not effectively control the infection. According to the literature, JEV infection can impair CD8^+^ T cell responses to broad-spectrum antigens by significantly altering antigen presentation 14. To investigate T cell responsiveness to broad antigens after P3 infection, the mice infected with JEV were then immunized with OVA. A CD8^+^ T cell killing assay in a mixed culture of sorted CD8^+^ T cells from immunized mice with OVA^+^ EL4 cells revealed that JEV infection weakened the ability of CD8^+^ T cells to kill target cells (Figure 4G). These results suggest that JEV infection induces exhaustion-like CD8^+^ T cells, which are associated with symptom onset and reduced cytokine production, as well as weakened immune responses to broad antigens.

### MDSCs induced exhaustion-like CD8^+^ T cells *in vitro*

**Figure 5:**
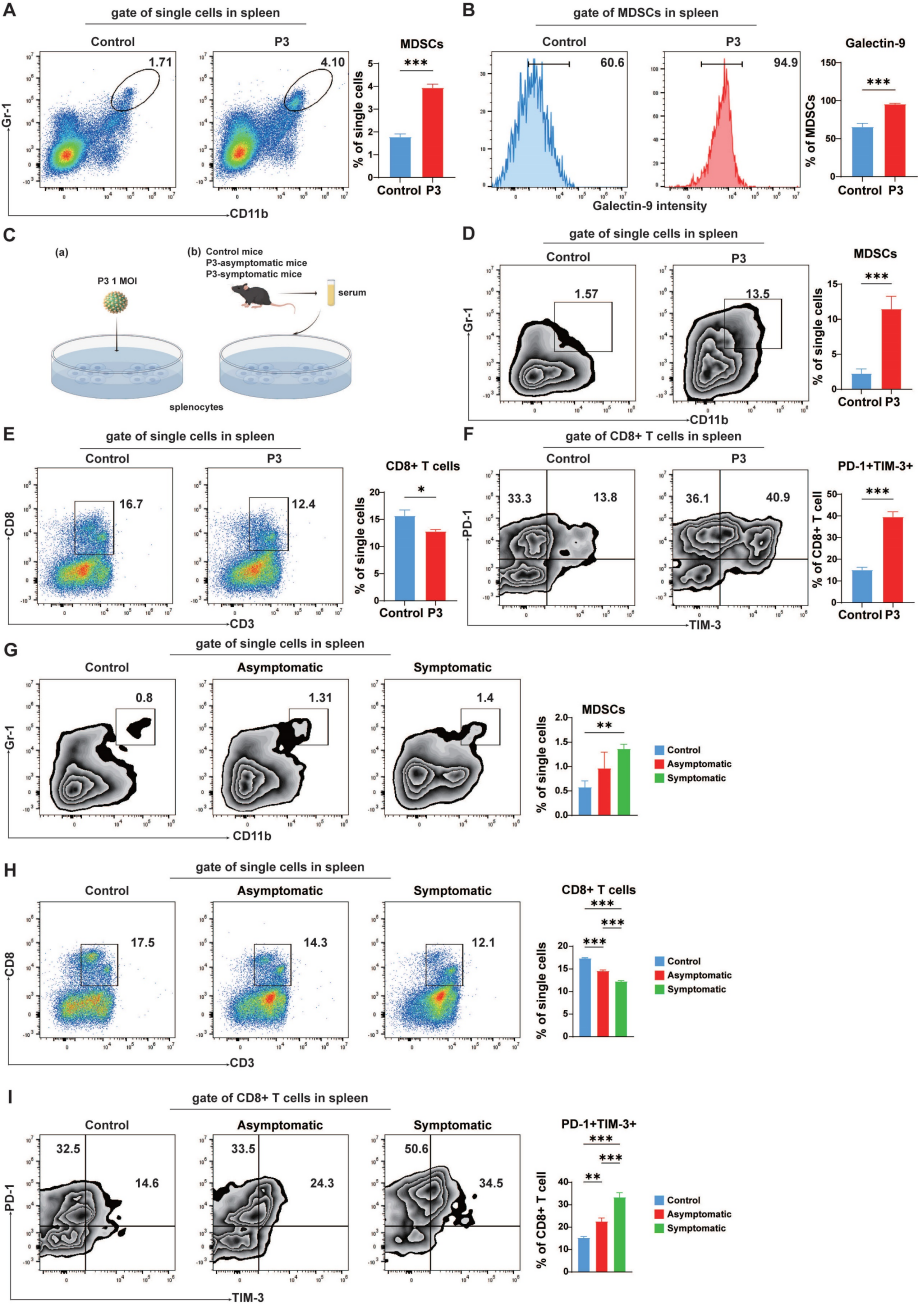
** The induction of exhaustion-like T cells *in vitro*.** C57BL/6 mice (female, 6-8 weeks old) were i.v. injected with 10^5^ PFUs of P3, and splenocytes were analyzed at 5 dpi. **(A)** Flow cytometry plots and frequencies of MDSCs in the spleens of control and P3-infected mice. **(B)** Flow cytometry histograms and frequencies of galectin-9^+^ MDSCs in the spleens of control and P3-infected mice. **(C)** Schematic diagram of *in vitro* splenocyte stimulation with P3 or serum from P3-infected mice. **(D, E, F)** Splenocytes were isolated from C57BL/6 mice, infected with P3 and analyzed by flow cytometry on 3 dpi. **(D)** Flow cytometry plots and frequencies of MDSCs in the splenocyte population. **(E)** Flow cytometry plots and frequencies of CD8^+^ T cells in the splenocyte population. **(F)** Flow cytometry plots and frequencies of PD-1^+^TIM-3^+^ cells within CD8^+^ T cells in the splenocyte population. **(G, H, I)** Splenocytes were isolated from C57BL/6 mice, and the serum from control, asymptomatic, and symptomatic mice was subsequently added to the medium. The splenocytes were analyzed by flow cytometry on day 3 after culture *in vitro*. **(G)** Flow cytometry plots and frequencies of MDSCs in the splenocyte population. **(H)** Flow cytometry plots and frequencies of CD8^+^ T cells in the splenocyte population. **(I)** Flow cytometry plots and frequencies of PD-1^+^TIM-3^+^ cells within CD8^+^ T cells in the splenocyte population. Representative results from repeated experiments are shown. The data are presented as arithmetic means ± SEMs. *, *P* < 0.05; **, *P* < 0.01; ***, *P* < 0.001.

JEV infection increased the number of MDSCs in the spleen (Figure 5A). An increase in galectin-9 on MDSCs, a TIM-3 ligand, could mediate T cell exhaustion in the context of malignancy 38. To assess whether JEV-induced MDSCs correlate with the emergence of exhaustion-like T cells after JEV infection, galectin-9 expression in MDSCs in the spleen was evaluated. Post-JEV infection, a significant increase in galectin-9 expression was observed on the cell surface of MDSCs (Figure 5B). As shown in Figure 5C (a), flow cytometry analysis of isolated murine splenic cells following JEV infection *in vitro* revealed an increase in MDSCs (Figure 5D) and a corresponding decrease in CD8^+^ T cells (Figure 5E), with notable upregulation of PD-1 and TIM-3 expression on CD8^+^ T cells (Figure 5F), mirroring the changes observed *in vivo* post-infection. Bat3/Bag6 has been reported to bind to the cytoplasmic domain of TIM-3 and inhibit signaling through this receptor 39. The expression of Bag6 in the two subsets of CD8^+^ T cells was assessed using flow cytometry. Both the percentage and MFI of Bag6 were significantly lower in PD-1^+^TIM-3^+^CD8^+^ T cells compared to PD-1^+^TIM-3^-^ cells, suggesting that the downstream inhibitory signaling pathway of TIM-3 may be activated (Supplementary Figure 3).

In addition to persistent antigenic stimulation, various soluble factors significantly contribute to T cell exhaustion. Following infection with JEV, notable upregulation of various cytokines, specifically interleukin-10 (IL-10), which MDSCs produce, is significantly elevated 11. Concurrently, the soluble form of galectin-9 is capable of downregulating the immune response 40, indicating its role beyond cell surface interactions. To investigate the presence of soluble factors capable of inducing exhaustion-like T cells, we added serum from both asymptomatic and symptomatic JEV-infected mice to splenic cell cultures *in vitro* (Figure 5C (b)). Serum from symptomatic JEV-infected mice led to an increase in MDSCs (Figure 5G) but a decrease in CD8^+^ T cells (Figure 5H), as well as upregulation of PD-1 and TIM-3 expression in CD8^+^ T cells (Figure 5I). Notably, stimulation with P3-infected serum primarily increased PD-1 expression, with less significant effects on TIM-3 expression (Figure 5I). Additionally, JEV-induced soluble factors (IL-10 and soluble galectin-9) played a similar role in promoting the development of exhaustion-like CD8^+^ T cells in splenic cells (Supplementary Figure 4). These findings suggest that JEV infection increased the proportion of MDSCs in the spleen with increased galectin-9 and IL-10 expression, which facilitated the generation of exhaustion-like T cells.

### Blockade of TIM-3 mitigated MDSC-induced exhaustion-like T cells

**Figure 6:**
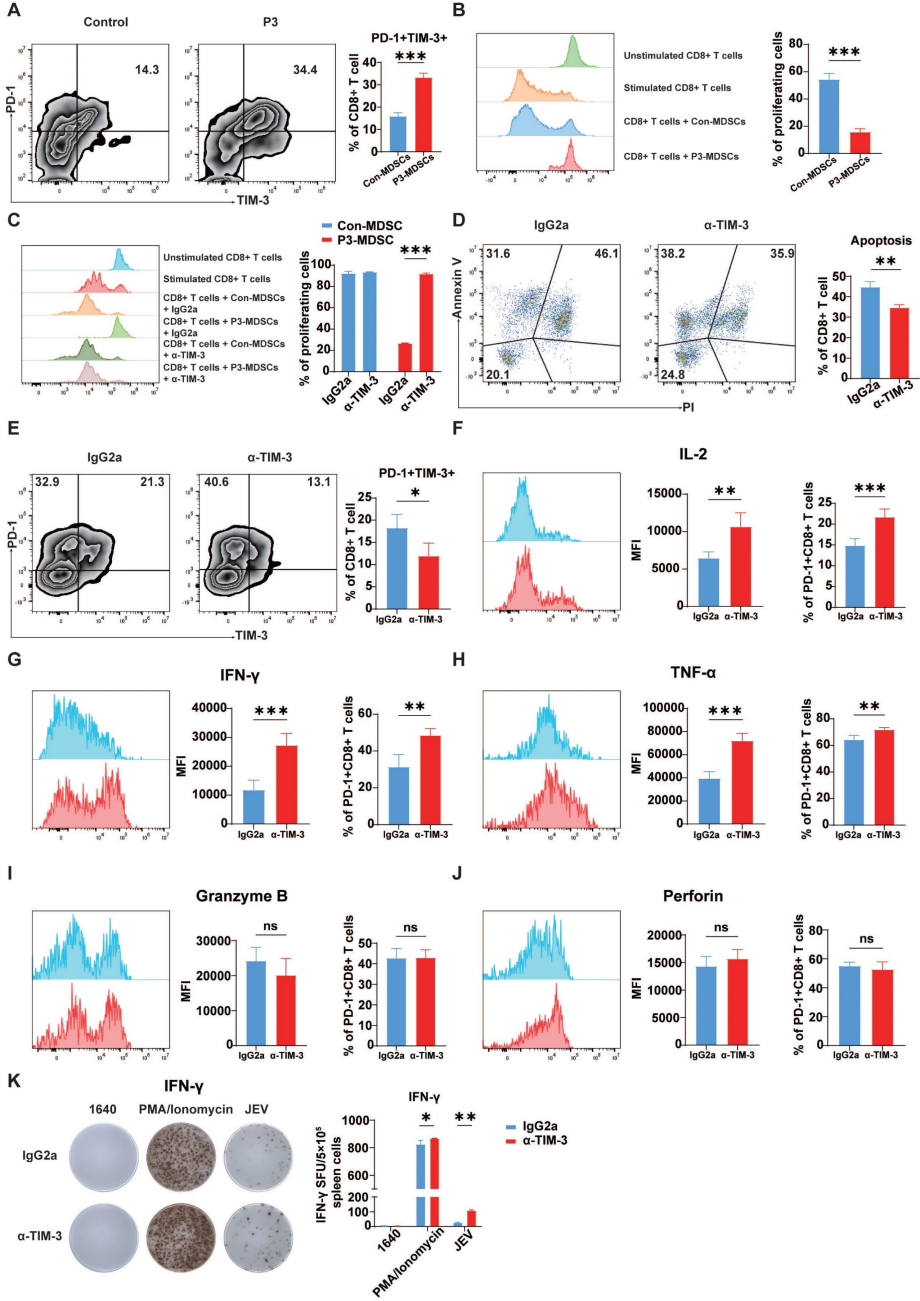
** α-TIM-3 treatment enhanced CD8^+^ T cell effector function *in vitro* and *in vivo*. (A, B)** MDSCs were sorted from the spleens of control and P3-infected mice and then cultured with CD8^+^ cells at a ratio of 1:1.** (A)** The frequencies of PD-1^+^TIM-3^+^ cells among CD8^+^ T cells were analyzed after 3 days. Flow cytometry plots (left) and frequencies (right) of PD-1^+^TIM-3^+^ cells within CD8^+^ T cells in the coculture system.** (B)** The proliferation of CD8^+^ T cells was analyzed after 4 days of incubation. Flow cytometry histograms (left) and proliferation rates (right) of CD8^+^ T cells in the coculture system.** (C)** Proliferation of CD8^+^ T cells in the presence of MDSCs from control and P3-infected mouse spleens with the addition of IgG2a or α-TIM-3. Flow cytometry histograms (left) and proliferation rates (right) of CD8^+^ T cells in the coculture system. **(D)** Apoptosis of CD8^+^ T cells in the presence of MDSCs from control and P3-infected mouse spleens with the addition of IgG2a or α-TIM-3. Flow cytometry plots (left) and frequency of late apoptotic CD8^+^ T cells (right) in the coculture system. **(E, F, G, H, I, J, K)** 100 μg of α-TIM-3 or IgG2a antibodies were i.p. injected every other day into control and P3-infected mice. Splenocytes were isolated from C57BL/6 mice and then analyzed via flow cytometry at 7 dpi. **(E)** Flow cytometry plots and frequencies of PD-1^+^TIM-3^+^ cells within CD8^+^ T cells in the spleens of mice infected with P3 and treated with α-TIM-3 or IgG2a antibodies. **(F, G, H, I, J)** Representative flow cytometry histograms (left), MFI (middle) and frequencies of positive cells (right) for IL-2, IFN-γ, TNF-α, granzyme B, and perforin staining in PD-1^+^CD8^+^ T cells are shown. **(K)** Frequencies of antigen-specific IFN-γ-secreting cells were assayed via IFN-γ ELISPOT using freshly isolated splenocytes at 7 dpi. The cells were stimulated with P3 peptides for 24 h. The bar graph shows the number of spot-forming units (SFUs) among 5×10^5^ splenocytes. Representative results from repeated experiments are shown. The data are presented as arithmetic means ± SEMs. ns, not significant; *, *P* < 0.05; **, *P* < 0.01; ***, *P* < 0.001.

Our previous studies demonstrated that JEV infection can promote MDSCs and limit the proliferation of CD8^+^ T cells via effector molecules such as Arg-1 41. MDSCs can induce T cell exhaustion in certain cancers and viral infections 38, 42. To confirm whether exhaustion-like CD8^+^ T cells were induced by MDSCs, pairs of control MDSCs and P3-infected MDSCs were cocultured with CD8^+^ T cells. Coculturing of CD8^+^ T cells with MDSCs generated from P3-infected mice dramatically increased PD-1 and TIM-3 expression on CD8^+^ T cells (Figure 6A). Additionally, JEV-induced MDSCs significantly inhibited CD8^+^ T cell proliferation, whereas MDSCs from control mice did not inhibit CD8^+^ T cell proliferation (Figure 6B). To determine whether TIM-3 inhibition prevents MDSC-induced suppression of T cell, CD8^+^ T cells were stimulated and cultivated with MDSCs for 4 days with the addition of TIM-3 antibody or isotype. Cell proliferation was assessed by flow cytometry. Blocking TIM-3 led to a considerable increase in CD8^+^ T cell proliferation when these cells were cocultured with JEV-induced MDSCs (Figure 6C). Galectin-9, a TIM-3 ligand, plays an important immune regulatory role by interacting with TIM-3 to induce apoptosis and inhibit T cell function in chronic HBV infection 43. Next, we investigated the correlation between JEV infection-induced apoptosis and TIM-3. Immunofluorescence staining of the spleen revealed a significant increase in TIM-3 expression on CD8^+^ T cells following JEV infection, which was consistent with the flow cytometry results (Supplementary Figure 5). Moreover, the percentage of TUNEL-positive TIM-3^+^CD8^+^ T cells was significantly greater than that of TIM-3^-^CD8^+^ T cells. We predicted that JEV infection induces apoptosis by increasing TIM-3 expression on CD8^+^ T cells. Thus, a TIM-3-blocking antibody was introduced into the coculture system comprising CD8^+^ T cells and JEV-induced MDSCs. α-TIM-3 reduced late apoptosis in CD8^+^ T cells while increasing the number of early apoptotic cells (Figure 6D).

Furthermore, the *in vivo* effects of TIM-3 blockade were assessed in a JEV-infected mouse model. Administration of α-TIM-3 significantly reduced the fraction of PD-1^+^TIM-3^+^CD8^+^ T cells in the spleen at 7 dpi. However, it greatly increased the percentage of PD-1^+^TIM-3^-^CD8^+^ T cells (Figure 6E). Administration of α-TIM-3 resulted in considerably increased MFI and the proportion of IL-2-positive PD-1^+^TIM-3^-^CD8^+^ T cells (Figure 6F). Additionally, the production of IFN-γ and TNF-α was markedly elevated after treatment with α-TIM-3 (Figure 6G & 6H). In contrast, no significant differences were observed in the expression of granzyme B or perforin, which are indicative of the ability to kill cells (Figure 6I & 6J). Since IFN-γ plays a crucial role in the anti-JEV response, the production of IFN-γ in mouse splenocytes was determined via ELISPOT (Figure 6K). The TIM-3 blocking antibody dramatically increased the expression of JEV-specific IFN-γ. Additionally, α-TIM-3 increased IFN-γ production in the positive control group treated with PMA/ionomycin. This result is consistent with the findings of the OVA^+^ cell killing assay (Figure 4G), suggesting that JEV, by upregulating TIM-3, not only inhibits the antigen-specific CD8^+^ T cell response but also impairs the ability of CD8^+^ T cells to respond to panantigens. In conclusion, JEV-induced MDSCs play an important role in producing exhaustion-like CD8^+^ T cells by increasing PD-1 and TIM-3 expression on CD8^+^ T cells. Blocking TIM-3 led to increased CD8^+^ T cell proliferation, reduced late apoptosis, and boosted the production of important cytokines such as IFN-γ and TNF-α.

### The depletion of MDSCs improved mouse survival by preventing the development of exhaustion-like T cells

**Figure 7:**
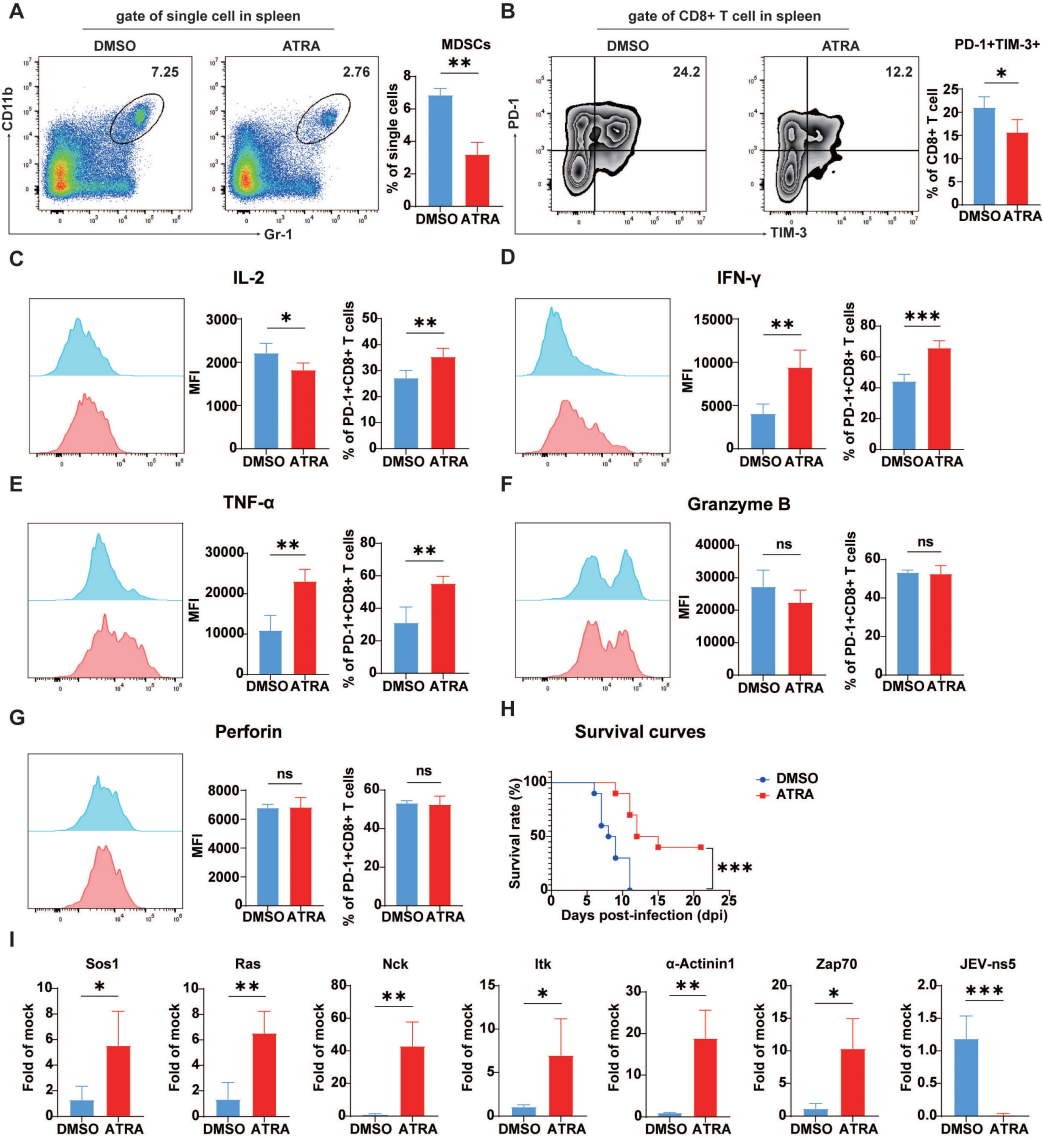
** Depletion of MDSCs rescued CD8^+^ T cells from exhaustion *in vivo* during P3 infection.** The mice were i.p. injected with ATRA (20 mg/kg/d) 3 days prior to P3 infection and then every 12 h until day 5. The splenic cells were analyzed via flow cytometry at 7 dpi.** (A)** A representative flow cytometry plot (left) and frequency (right) of MDSCs in the spleens of mice at 5 dpi. **(B)** Flow cytometry plots and frequencies of PD-1^+^TIM-3^+^ cells within CD8^+^ T cells in the splenocyte population. **(C, D, E, F, G)** Representative flow cytometry histograms (left), MFI (middle) and frequencies of positive cells (right) for IL-2, IFN-γ, TNF-α, granzyme B, and perforin staining in PD-1^+^CD8^+^ T cells are shown. **(H)** Survival curve of mice treated with ATRA or vehicle over a 21-day period of P3 infection (n=15). **(I)** The mRNA expression of TCR signaling-related genes and NS5 of P3 in P3-infected splenocytes was determined via real-time PCR at 7 dpi. Representative results from repeated experiments are shown. The data are presented as arithmetic means ± SEMs. ns, not significant; *, *P* < 0.05; **, *P* < 0.01; ***, *P* < 0.001.

*In vitro* studies have shown that JEV-induced MDSCs can suppress CD8^+^ T cell growth and induce exhaustion-like CD8^+^ T cells. To confirm whether these effects also occur *in vivo* MDSCs were depleted in mice using ATRA. This therapy dramatically decreased the number of splenic MDSCs in JEV-infected animals (Figure 7A). Concurrently, the fraction of splenic PD-1^+^TIM-3^+^CD8^+^ T cells was significantly reduced in MDSC-depleted animals (Figure 7B). Further functional analysis demonstrated that after MDSC elimination, the ratio of IL-2-positive cells among PD-1^+^CD8^+^ T cells increased significantly (Figure 7C). Additionally, both the MFI and positivity rate for IFN-γ and TNF-α were significantly elevated (Figure 7D & 7E). However, ATRA treatment did not alter the generation of granzyme B and perforin (Figure 7F & 7G). These findings were comparable with those obtained from the injection of TIM-3 blocking antibodies. Depletion of MDSCs also delayed illness onset and greatly boosted JEV-infected mouse survival rates (Figure 7H).

In chronic infections and tumor diseases, T cell exhaustion is primarily driven by impaired activation of the TCR signaling pathway. qPCR analysis revealed that ATRA-mediated removal of MDSCs significantly increased the expression of genes related to the TCR signaling pathway, Sos1, Ras, Nck, Itk, α-actinin1, Zap70 (Figure 7I), while promoting the viral clearance (Figure 7I, far right), suggesting that the JEV-induced inhibition of TCR signaling in CD8^+^ T cells was alleviated by depletion of MDSCs, leading to reduced exhaustion-like T cells.

In combination, JEV infection dramatically increased TIM-3 and PD-1 expression on CD8^+^ T cells, which was related to decreased cell function, impaired differentiation, and a worse prognosis in mice. *In vitro* and *in vivo* investigations demonstrated that MDSCs caused an exhaustion-like condition in CD8^+^ T cells, which may be relieved by inhibiting TIM-3. Furthermore, reducing the number of MDSCs led to a decrease in exhaustion-like T cells while significantly increasing infected mouse survival rates. These findings emphasize the critical function of MDSCs in inducing exhaustion-like CD8^+^ T cells during JEV infection, pointing to possible therapeutic routes for JE therapy.

## Discussion

T cell exhaustion is commonly regarded as a state exhibited in CD8^+^ T cells after persistent infections and malignant conditions. Recent investigations have demonstrated that an exhaustion-like phenotype can arise during the acute phase of various illnesses 23-25, 44, 45. In this study, we showed that JEV infection could cause an exhaustion-like status in CD8^+^ T cells, allowing viral immune evasion. Our findings showed that JEV infection dramatically reduced the number of CD8^+^ T cells, with activation of PD-1 and TIM-3 on these cells. CD8^+^ T cells that coexpressed PD-1 and TIM-3 demonstrated functional exhaustion, which was linked to more severe clinical symptoms in JEV-infected mice. Further analysis demonstrated that JEV-generated MDSCs induced an exhaustion-like phenotype in CD8^+^ T cells via the Galectin-9/TIM-3 pathway. MDSC elimination restored CD8^+^ T cell responses and increased infected mice's survival rates. Blocking TIM-3 also reinforced CD8^+^ T cell functionality by rescuing the expression of IFN-γ and TNF-α. These findings shed light on the processes by which JEV diminishes CD8^+^ T cell function, as well as providing experimental evidence that T cell exhaustion might occur during JEV infection. This study emphasizes the importance of evaluating T cell exhaustion in the setting of acute viral infections and reveals possible therapeutic targets for improving immune responses to JEV.

Flavivirus infections are usually considered to effectively activate CD8^+^ T cells, which are crucial for eliminating viral infections 46, 47. However, recent research has indicated that JEV induces profound immunosuppression by compromising the function of DCs, thereby significantly affecting CD8^+^ T cell responses. The number of CD8α^+^CD11c^+^ DCs, which are responsible for initiating CD8^+^ T cell responses, is reduced during JEV infection, resulting in inadequate activation and a subsequent decrease in antigen presentation 48. This observation is consistent with our findings, which revealed a considerable decrease in the proportion of CD8^+^ T cells during the early stages of JEV infection, along with insufficient activation of CD8^+^ T cells in JEV-infected mice. Similar immune suppression has been described in DENV infections, where CD8^+^ T cells exhibit reduced responsiveness due to the inhibition of costimulatory molecules and MHC molecules on DCs 49. In WNV-infected mice, T cell priming is similarly impaired by inhibition of DC activation 50. These findings underscore a common mechanism among flaviviruses, which subvert host immune responses by inhibiting the immune response capability of CD8^+^ T cells.

T cell exhaustion is a well-known phenomenon in chronic viral infections and cancers, characterized by a progressive loss of effector properties, decreased proliferative ability, and persistent expression of inhibitory receptors. Chronic infections with viruses such as HBV, HCV, and HIV often affect CD8^+^ T cell responses and differentiation through multiple mechanisms, leading to T cell exhaustion, which severely compromises the ability of the host to control the infection and affects disease prognosis 21, 22, 51-54. In contrast, during acute infections, CD8^+^ T cells normally proliferate rapidly and then contract once the pathogen is eliminated. However, recent research has demonstrated that CD8^+^ T cells in individuals with acute infections, such as COVID-19, exhibit an exhaustion profile similar to that observed in individuals with persistent viral infections. Severe COVID-19 patients present high levels of T cell exhaustion markers, which are correlated with disease severity and poor outcomes 23, 45, 55, 56. Similarly, single-cell sequencing of severe dengue patients revealed considerable upregulation of coinhibitory molecules and inadequate expression of effector molecules on CD8^+^ T cells. Although these findings are congruent with the gene expression profiles of exhausted T cells, functional verification of CD8^+^ T cells has yet to be performed 25.

CD8^+^ T cells activated during the acute phase of viral infection often express PD-1 and other inhibitory receptors 57-59, complicating the distinction between true T cell exhaustion and transient immune regulation. Given that JEV infection is also an acute infection disease, with the viral infection cycle in mice typically concluding within 21 days 60, we chose this observation period to evaluate the phenotype of CD8^+^ T cells to effectively capture the dynamics of CD8^+^ T cell immune responses during JEV infection and assessing the potential development of T cell exhaustion in acute viral infections. Our findings revealed a significant increase in PD-1 and TIM-3 coexpression on CD8^+^ T cells throughout the 21-day in JEV-infected mice, indicating an exhaustion-like phenotype 19, 21, 32, 51. To determine whether this subset of CD8^+^ T cells is prone to exhaustion, a functional investigation of CD8^+^ T cells was conducted in JEV-infected mice. Throughout the 21-day infection period, the levels of cytokines, such as IL-2, TNF-α, and IFN-γ were much lower in PD-1^+^TIM-3^+^ exhaustion-like CD8^+^ T cells than in PD-1^+^TIM-3^-^CD8^+^ T cells, which are recognized as activated cells. Furthermore, the quantity and functional state of exhaustion-like CD8^+^ T cells are crucial factors associated with infection outcomes of JEV. These results are similar to the findings of T cell exhaustion in cancer and chronic viral infections. Importantly, similar mechanisms have been observed in other flavivirus infections. In WNV infections, the elevated expression of TIM-3 leads to decreased IFN-γ output and poor viral clearance 61. In patients with DENV infection, increased levels of TIM-3 also impair IFN-γ secretion by γδ T cells 62. In light of this, TIM-3 appears to serve as a critical regulatory molecule that inhibits both cytokine production and effector function in flavivirus infection. Beyond impairing cytokine output, TIM-3 also influences the proliferative capacity of T cells. While both populations retain the capacity to proliferate, high TIM-3 expression appears to limit the proliferative ability of CD8^+^ T cells during JEV infection, as fewer PD-1^+^TIM-3^+^ cells were actively proliferating compared to PD-1^+^TIM-3^-^ cells. Despite this reduction, PD-1^+^TIM-3^+^ cells do not lose their proliferative capacity, which is consistent with findings from other studies. This observation parallels results from a recent study on ovarian cancer, which demonstrated that PD-1^+^TIM-3^+^CD8^+^ TILs retained the proliferative potential but exhibited impaired functionality, leading to poor clinical outcomes 63. In addition, single-cell sequencing data from tumor models have shown that exhausted T cells continue to express Ki-67, indicating that despite their functional exhaustion, these cells maintain the capacity for proliferation 64. However, high TIM-3 expression does not impair the degranulation capabilities of CD8^+^ T cells during JEV infection, which differs from the traditional characteristics of exhausted T cells but is similar to reports in mild or asymptomatic COVID-19 patients 65. Therefore, we chose the term "exhaustion-like" to more precisely define the state of CD8^+^ T cells in the context of JEV infection, reflecting both their shared characteristics with traditional exhausted T cells and the distinct aspects arising from the acute infection setting.

TIM-3 has both costimulatory and coinhibitory effects on CD8^+^ T cells and regulates immunological responses in a context-dependent manner. When activated, TIM-3 can operate as a costimulatory factor, promoting the MEK-ERK and PI3K-Akt signaling pathways and therefore increasing cytotoxic functions 66. However, in chronic infections, the interaction of TIM-3 with its ligands, such as galectin-9, alters its function toward immunological suppression. This interaction causes the release of the adaptor protein Bat3 from TIM-3, facilitating the inhibitory signaling that suppresses TCR activation and function 39, 67. ERK signaling is crucial for the activation of degranulation functions in immune cells 68, 69, and the release of Bat3 results in a significant reduction in IFN-γ expression 39. Consequently, during the early stages of JEV infection, CD8^+^ T cells retain their cytotoxic activity, whereas Bat3 release concurrently suppresses cytokine production, resulting in CD8^+^ T cells exhibiting both activation and exhaustion phenotypes simultaneously. During viral infections, TCR signaling is required to initiate and sustain T cell responses. Sustained antigen exposure and inflammatory signals can lead to alterations in TCR signaling pathways, resulting in the induction of coinhibitory molecule expression and subsequent T cell exhaustion 70-72. These coinhibitory molecules can further suppress TCR signal activation 73. The binding of the ligand to TIM-3 prevents Bat3 from interacting with activated Lck, thereby regulating downstream TCR signaling 39. In addition to TIM-3, PD-1 signaling also inhibits the activation of the PI3K-Akt pathway, further suppressing the ITAM pathway of TCR signaling 74. Our work revealed that JEV infection disrupted TCR signaling, resulting in decreased production of important cytokines, such as IFN-γ and TNF-α, by CD8^+^ T cells. Inadequate activation of TCR signaling is the underlying cause of T cell exhaustion, which in turn results in a more pronounced degree of malfunction. Further research into the underlying signaling system is needed for a thorough understanding of T cell activation and exhaustion during acute viral infections.

MDSCs are essential immunosuppressive cells that cause T cell exhaustion during chronic viral infections and tumor development 38, 42. Our findings demonstrated that JEV-induced MDSCs express significant quantities of galectin-9, which interacts with TIM-3 on CD8^+^ T cells, leading to the formation of exhaustion-like T cells and apoptotic cell death. In acute viral infections, galectin-9 has been shown to influence virus-specific CD8^+^ T cell responses. Knocking down galectin-9 or employing galectin-9 inhibitors can improve the responses of these virus-specific CD8^+^ T cell populations 75-77. When T cells are exhausted, blocking TIM-3 or altering the TIM-3/Galectin-9 connection has been demonstrated to restore T cell activity and improve immune responses in chronic viral infections and cancer models 78-81. In this study, the administration of a TIM-3 blocking antibody dramatically increased the proliferation of CD8^+^ T cells in coculture with MDSCs. It also restored the expression of inflammatory cytokines, specifically IFN-γ, in CD8^+^ T cells. These findings indicate that targeting the TIM-3/Galectin-9 link between CD8^+^ T cells and MDSCs could be an effective treatment strategy for reversing the exhaustion-like status of CD8^+^ T cells and boosting antiviral immunity in JEV infection.

In patients with tumors, upregulated TIM-3 on the surface of T cells can interact with MDSCs, inhibiting T cell proliferation and further contributing to immunosuppression 82, 83. Therefore, as important immune regulatory cells involved in the early stage of JEV infection, the interaction of MDSCs with CD8^+^ T cells is pivotal for triggering T cell exhaustion. TIM-3 is the key receptor on CD8^+^ T cells coupled with galectin-9 to mediate exhaustion. Blocking TIM-3 significantly reinforced CD8^+^ T cell functionality. Moreover, TIM-3 is also present in myeloid cells, where its expression suppresses inflammatory responses and antigen presentation 84, 85. In tumor-bearing mice, blocking TIM-3 can significantly enhance the cytotoxic function of CD8^+^ T cells by restoring the antigen-presenting function of dendritic cells 86. Thus, targeting TIM-3 can directly activate CD8^+^ T cell function and indirectly activate CD8^+^ T cells by regulating myeloid cell function. Furthermore, during JEV infection, MDSCs inhibit T cell responses through various mechanisms, such as reactive oxygen species, nitric oxide, and immunosuppressive cytokines, such as IL-10 11, 41. Consequently, in JEV P3-infected mice, the depletion of MDSCs more effectively restored the cytokine-producing capacity of CD8^+^ T cells than the blockade of TIM-3.

## Conclusion

In summary, our findings show that JEV causes an exhaustion-like status in CD8^+^ T cells, as shown by increased coexpression of PD-1 and TIM-3, impairing immunological function and leading to more severe disease outcomes. MDSCs have been identified as mediators of CD8^+^ T cell exhaustion via the TIM-3/Galectin-9 pathway, which further inhibits TCR signaling pathway activation and, as a result, suppresses CD8^+^ T cell inflammatory factor production. Blocking TIM-3 or decreasing the number of MDSCs restores CD8^+^ T cell function and increases survival in JEV-infected mice. These findings indicate that targeting MDSCs or inhibiting pathways such as TIM-3 could be effective therapeutic strategies for increasing antiviral immunity and improving outcomes in Japanese encephalitis patients.

## Supplementary Material

Supplementary figures.

## Figures and Tables

**Figure 8:**
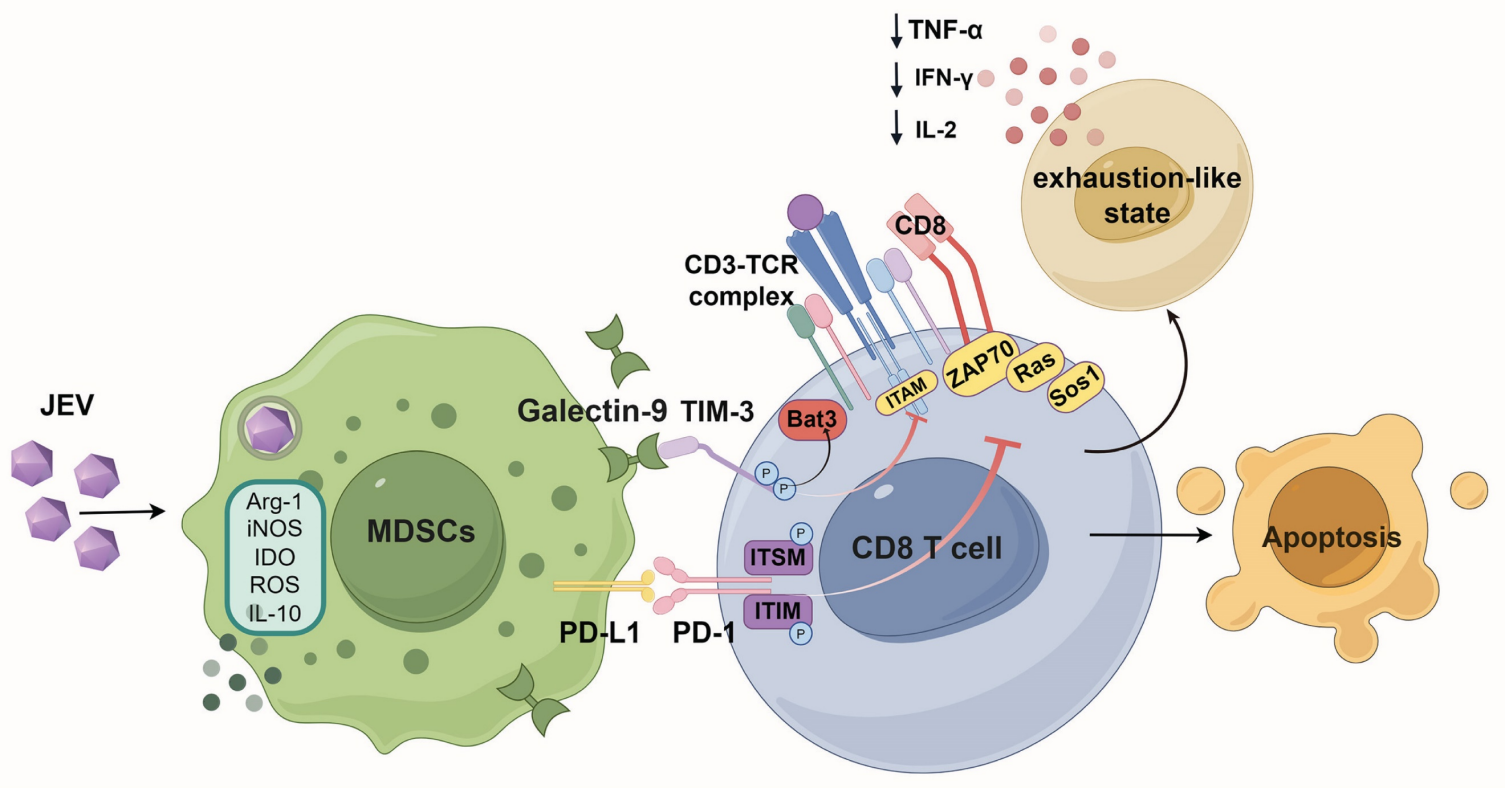
** Schematic diagram of how JEV infection induces exhaustion-like CD8^+^ T cells through MDSCs.** Japanese encephalitis virus (JEV) infection promotes the activity of myeloid-derived suppressor cells (MDSCs), which subsequently secrete immunosuppressive factors such as arginase-1 (Arg-1), inducible nitric oxide synthase (iNOS), indoleamine 2,3-dioxygenase (IDO), reactive oxygen species (ROS), and interleukin-10 (IL-10). These factors collectively inhibit the function of CD8^+^ T cells. The interaction between galectin-9 and TIM-3 on CD8^+^ T cells, along with the interaction of PD-L1 on MDSCs with PD-1 on CD8^+^ T cells, suppresses TCR signaling. This suppression results in the downregulation of critical signaling molecules such as Ras, Sos1 and ZAP70, further inhibiting T cell activation. Consequently, there is decreased production of cytokines such as IL-2, IFN-γ, and TNF-α. This immunosuppressive cytokine environment fosters an exhaustion-like status in CD8^+^ T cells, ultimately leading to apoptosis.

## Abbreviations

BBB: blood-brain barrier
CTLs: cytotoxic T lymphocytes
DCs: dendritic cells
DENV: dengue virus
DMEM: Dulbecco's modified Eagle's medium
dpi: days post infection
HCV: hepatitis C virus
HIV: human immunodeficiency virus
i.p.: intraperitoneally
i.v.: intravenously
IFN: interferon
IL: interleukins
JE: Japanese encephalitis
JEV: Japanese encephalitis virus
MDSCs: myeloid-derived suppressor cells
MOI: multiplicity of infection
PFUs: plaque-forming units
Tcm: central memory T cells
Tem: effector memory T cells
Tn: naive T cells
TNF: tumor necrosis factor
WNV: west nile virus
ZIKV: Zika virus
