# Supplementary material for: Myeloid-Derived Suppressor Cells Induce Exhaustion-Like CD8+ T Cells during JEV Infection
Source: Int J Biol Sci. 2024 Nov 4;20(15):5959–78. doi: 10.7150/ijbs.102372 (PMC11628328; doi:10.7150/ijbs.102372)
Supplement: Supplementary file 1 — Supplementary figures. [file ijbsv20p5959s1.pdf]

Supplementary Figure 1

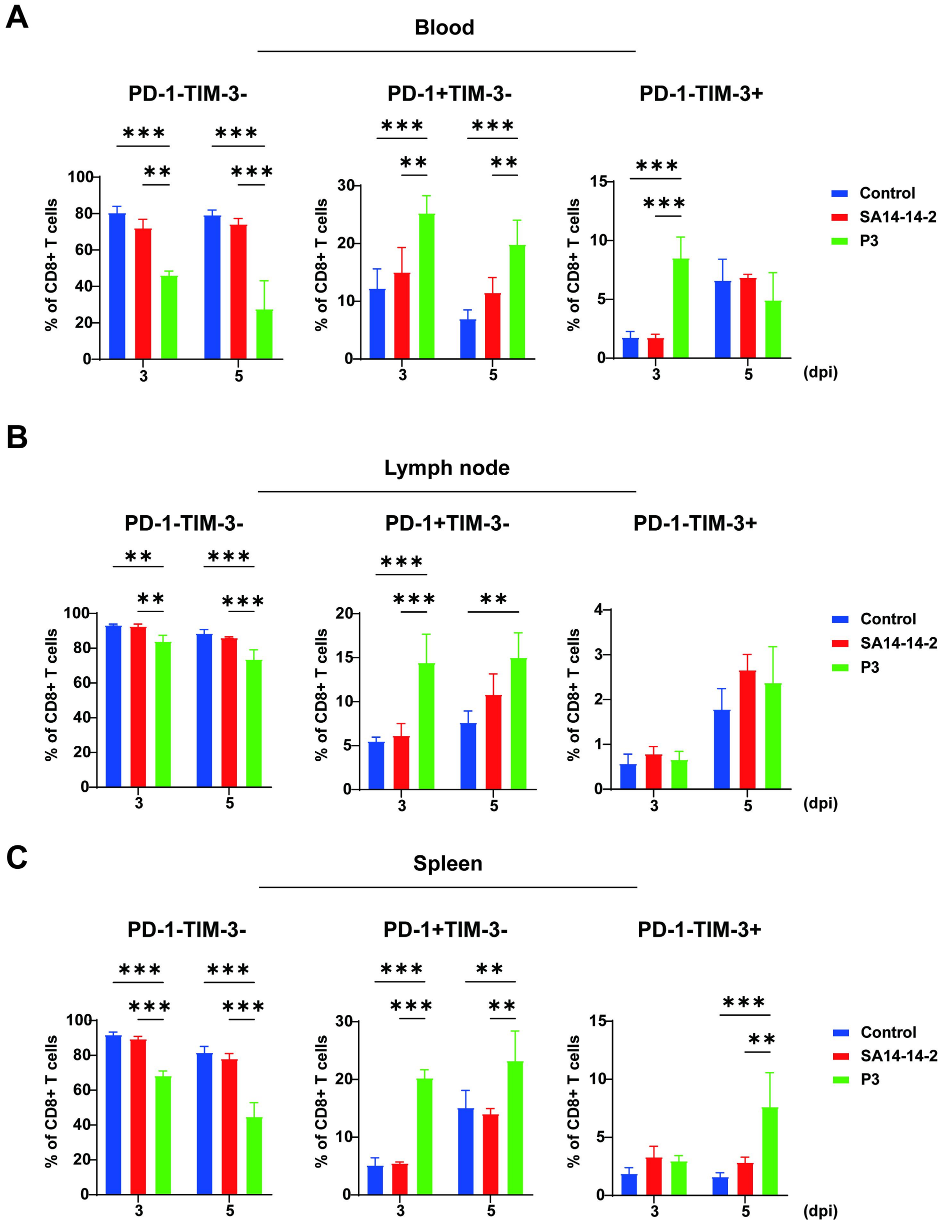

Supplementary Figure 1 (related to Figure 1).

Statistical graphs depicting the percentages of PD-1$^-$TIM-3$^-$, PD-1$^+$TIM-3$^-$, and PD-1$^-$TIM-3$^+$ cells within CD8$^+$ T cells in: (A) Peripheral blood, (B) Lymph nodes and (C) Spleen. Representative results from repeated experiments are shown. The data are presented as arithmetic means ± SEMs. *, $P < 0.05$; **, $P < 0.01$; ***, $P < 0.001$.

Supplementary Figure 2

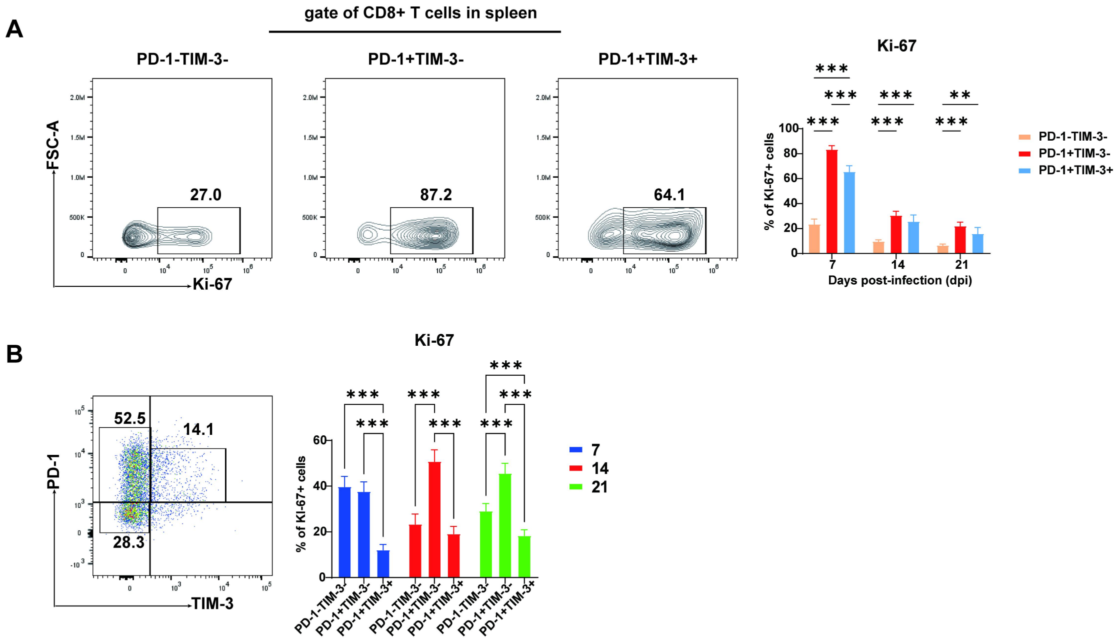

Supplementary Figure 2 (related to Figure 2).

(A) Representative flow cytometry plots (left) and statistical graph of the proportion of Ki-67 positivity rate in different $CD8^+$ T cell subsets (right) are shown. (B) A representative flow cytometry plot and frequencies of P3-infected mouse spleens gated on Ki-67^+ cells at 7, 14, and 21 dpi were analyzed for different $CD8^+$ subsets. Representative results from repeated experiments are shown. The data are presented as arithmetic means ± SEMs. *, $P < 0.05$; **, $P < 0.01$; ***, $P < 0.001$.

Supplementary Figure 3

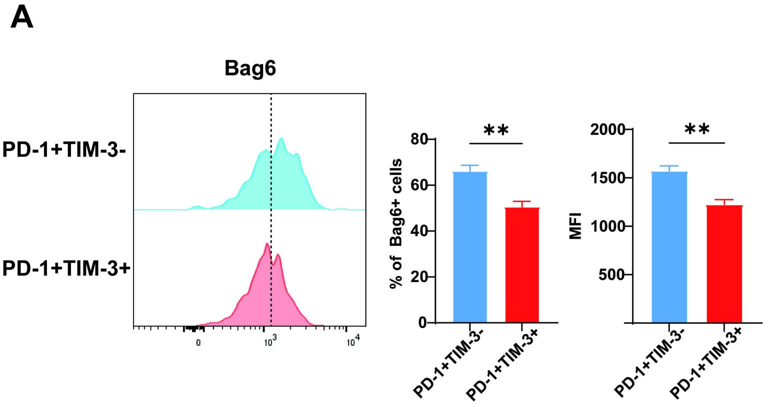

Supplementary Figure 3 (related to Figure 5).

(A) Representative flow cytometry histograms (left), frequency (middle) and MFI (right) of Bag6 staining in $CD8^{+}$ T cells are shown. Representative results from repeated experiments are shown. The data are presented as arithmetic means ± SEMs. *, $P < 0.05$; **, $P < 0.01$; ***, $P < 0.001$.

Supplementary Figure 4

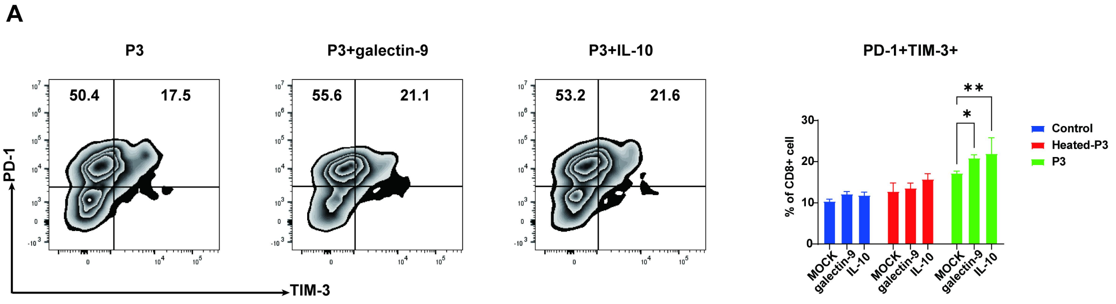

Supplementary Figure 4 (related to Figure 5).

Galectin-9 (10 ng/ml) and IL-10 (10 ng/ml) were added to the culture medium of splenocytes infected with P3 or heated-P3 (MOI of 1). **(A)** Flow cytometry plots and frequencies of PD-1$^-$TIM-3$^-$, PD-1$^+$TIM-3$^-$, and PD-1$^+$TIM-3$^+$ cells within CD8$^+$ T cells in the P3-infected splenocyte population. Representative results from repeated experiments are shown. The data are presented as arithmetic means ± SEMs. *, $P < 0.05$; **, $P < 0.01$; ***, $P < 0.001$.

Supplementary Figure 5

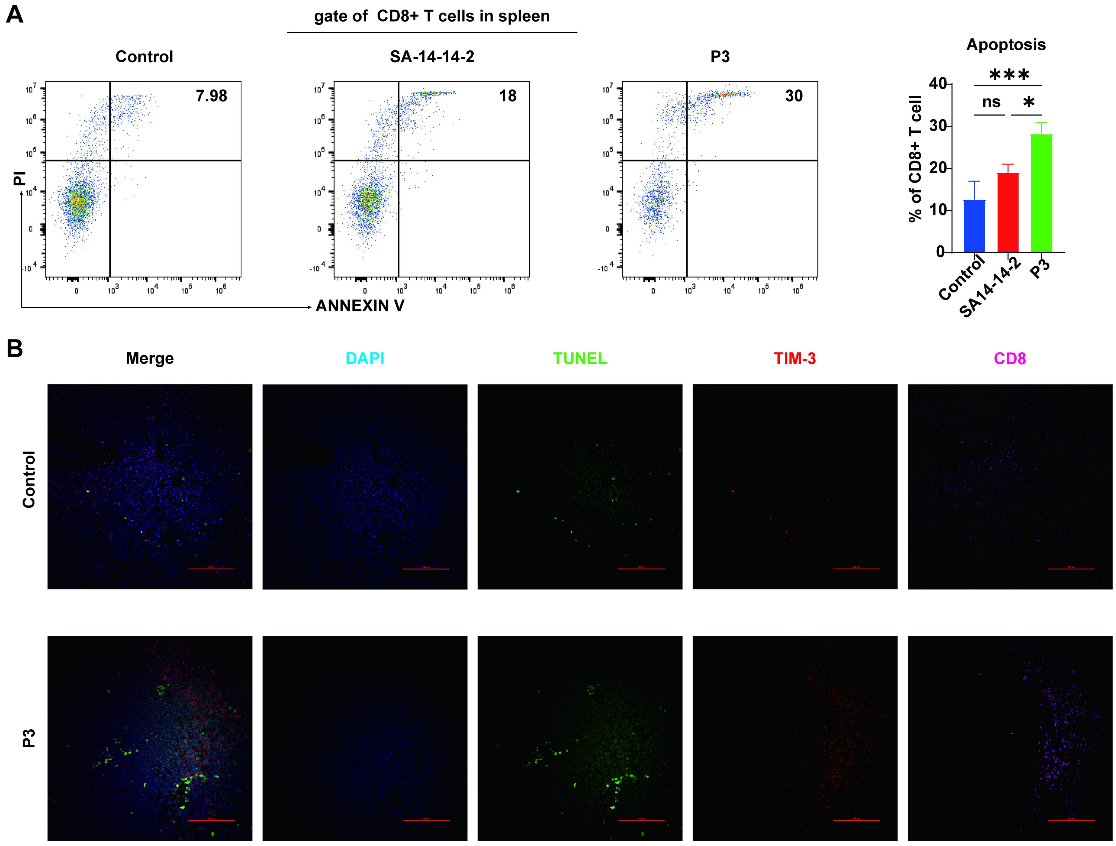

Supplementary Figure 5 (related to Figure 6).

(A) Representative flow cytometry plots and frequencies of CD8^{+} T cells from the spleens of control, SA14-14-2 and P3-infected mice were stained with PI and Annexin V. Representative results from repeated experiments are shown. The data are presented as arithmetic means ± SEMs. ns, not significant; *, $P < 0.05$; **, $P < 0.01$; ***, $P < 0.001$.

(B) TUNEL staining results of spleens from control and P3-infected mice. The representative DAPI (blue), TUNEL (green), TIM-3 (red), and CD8 (pink) staining results of spleens from mice in the control and P3-infected groups; scale bar = 100 μm.
